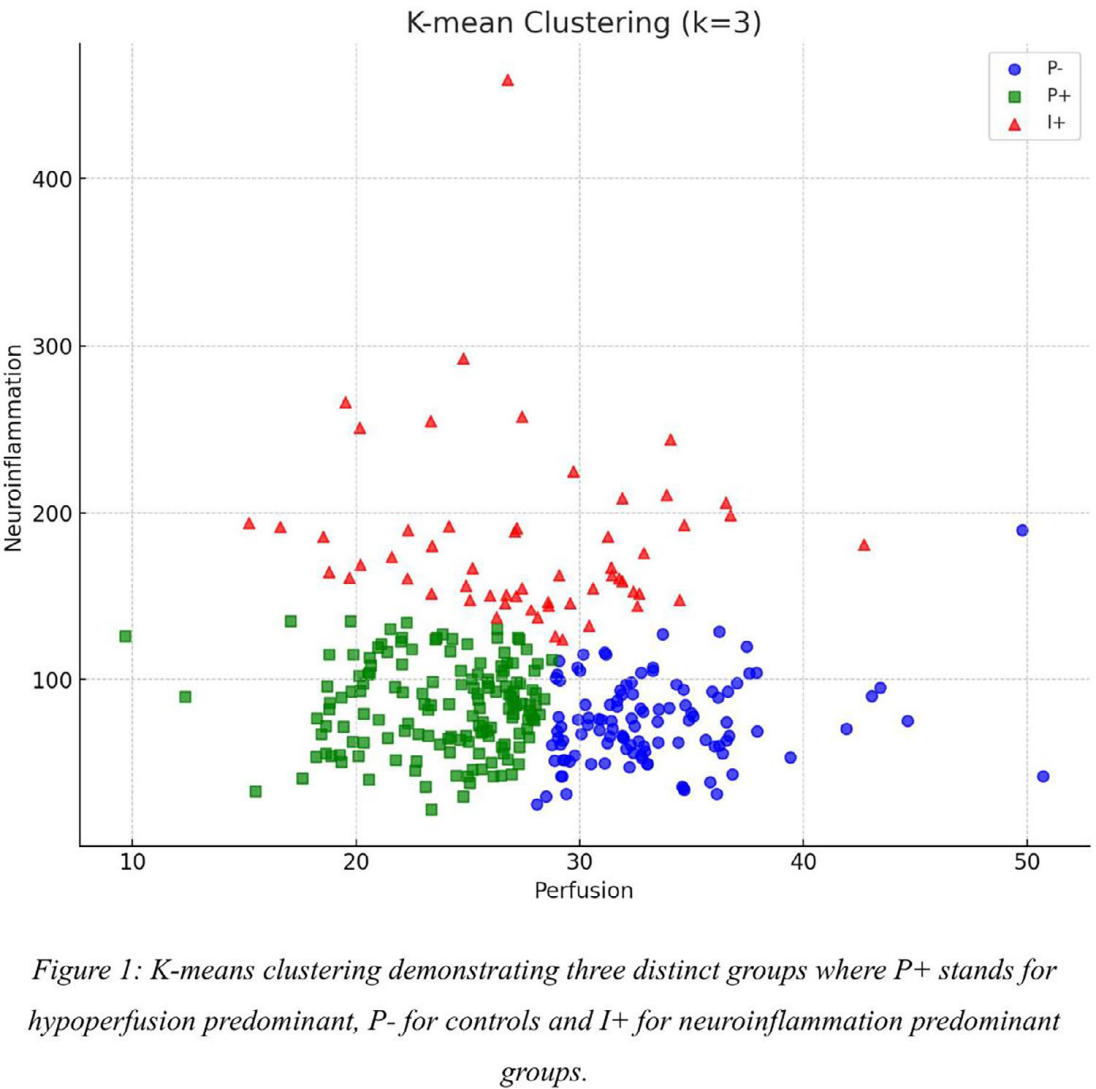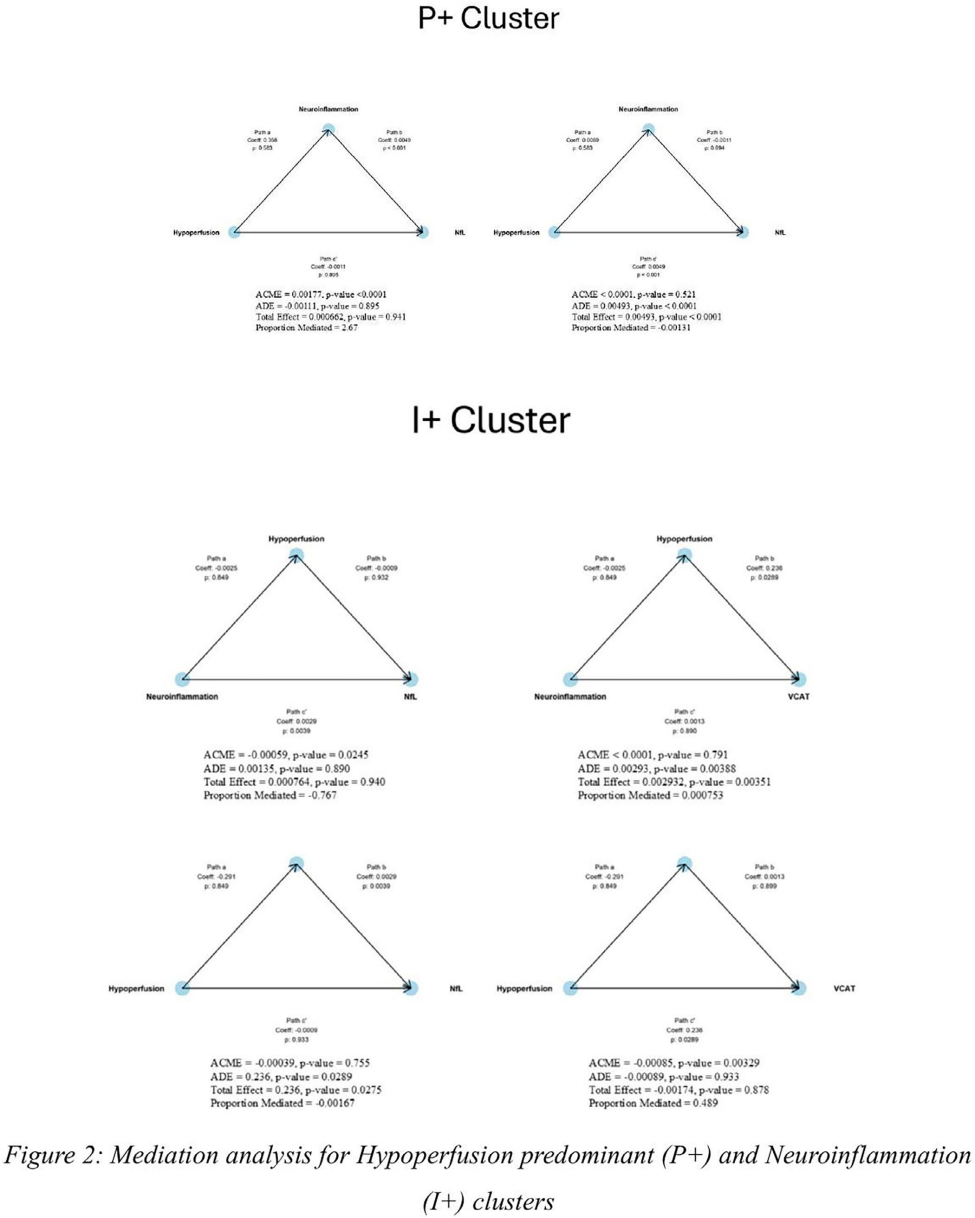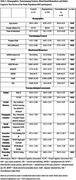# Interplay of Hypoperfusion and Neuroinflammation: Effects on Biomarkers and Cognition in Mild Cognitive Impairment

**DOI:** 10.1002/alz70857_105462

**Published:** 2025-12-24

**Authors:** Jia Dong James Wang, Yi Jin Leow, Ashwati Vipin, Gurveen Kaur Sandhu, Justin Jit Hong Ong, Seyed Ehsan Saffari, Nagaendran Kandiah

**Affiliations:** ^1^ Lee Kong Chian School of Medicine, Nanyang Technological University, Singapore, Singapore, Singapore; ^2^ nil, nil, nil, Nicaragua; ^3^ Lee Kong Chian School of Medicine, Nanyang Technological University, Singapore, Singapore; ^4^ Dementia Research Centre (Singapore), Lee Kong Chian School of Medicine, Nanyang Technological University, Singapore, Singapore; ^5^ National Neuroscience Institute, Singapore, Singapore; ^6^ Health Services and Systems Research, Duke‐NUS Medical School, Singapore, Singapore, Singapore; ^7^ National Healthcare Group, Singapore, Singapore; ^8^ Duke‐NUS Medical School, National University of Singapore, Singapore, Singapore

## Abstract

**Background:**

Cerebral hypoperfusion and neuroinflammation are interrelated pathophysiological processes contributing to mild cognitive impairment (MCI), often perpetuating a self‐reinforcing cycle of oxidative stress and inflammation. This study investigates distinct cognitive and plasma biomarker profiles in a Southeast Asian cohort to differentiate vascular and inflammatory‐driven cognitive impairments.

**Method:**

Participants with MCI were selected from the Biomarkers and Cognition Study, Singapore based on the NIA‐AA criteria. Using k‐means clustering, they were categorized into hypoperfusion‐predominant (*p* +), neuroinflammation‐predominant (I+), and control (*p*‐) groups based on MRI arterial spin labelling global grey matter perfusion and Glial Fibrillary Acidic Protein (GFAP) levels. Cognitive performance and biomarker profiles were compared across the three clusters (i.e. P+, I+, *p*‐). Mediation analysis examined the interactions and interdependencies between hypoperfusion and neuroinflammation.

**Result:**

The P+ group exhibited significantly elevated Neurofilament Light Chain (NfL) levels compared to the *p*‐ group (*p* = 0.0395, Mean Difference (MD)=0.211). Similarly, the I+ group demonstrated increased NfL levels (*p* = 0.0001, MD=0.279), elevated Oligomeric Amyloid‐Beta (OAB) levels (*p* = 0.0228, MD=0.116), a reduced Amyloid‐Beta 42/40(Aβ42/40) ratio (*p* = 0.0008, MD=‐0.0086), and lower global cognitive performance as assessed by the Visual Cognitive Assessment Tool (VCAT) (*p* = 0.0006, MD=‐1.83) compared to P+ group. When comparing the I+ and *p*‐ groups, the I+ group exhibited significantly higher NfL levels (*p* <0.0001, MD=0.491), elevated OAB levels (*p* = 0.0079, MD=0.139), a reduced Aβ42/40 ratio (*p* = 0.0031, MD=‐0.0081), and lower VCAT scores (*p* = 0.0011, MD=‐1.85).

Mediation analysis revealed that neuroinflammation mediated the relationship between hypoperfusion and elevated NfL levels in the P+ group (ACME=0.00177, *p* <0.0001). In contrast, reduced perfusion mediated the effect of neuroinflammation on global cognitive decline (VCAT) in the I+ group (ACME=‐0.00085, *p* = 0.00329).

**Conclusion:**

These findings underscore distinctive biomarkers that differentiate neuroinflammation‐predominant and hypoperfusion‐predominant MCI. They further reveal the intricate interplay between hypoperfusion and neuroinflammation, highlighting their interdependence and combined contributions to MCI pathology. These results emphasize the need for personalized therapeutic strategies addressing the dual pathologies of cerebrovascular and inflammatory dysfunctions, particularly in populations with region‐specific variations in MCI pathology.